# Endotoxins Induced ECM-Receptor Interaction Pathway Signal Effect on the Function of MUC2 in Caco2/HT29 Co-Culture Cells

**DOI:** 10.3389/fimmu.2022.916933

**Published:** 2022-06-10

**Authors:** Wenxiang Hu, Ping Feng, Mingming Zhang, Tian Tian, Shengxiang Wang, Baoyu Zhao, Yajie Li, Shuo Wang, Chenchen Wu

**Affiliations:** ^1^ College of Animal Veterinary Medicine, Northwest A & F University, Yanling City, China; ^2^ College of Life Sciences, Yulin University, Yulin, China

**Keywords:** lipopolysaccharide (LPS), Caco2/HT-29 cells, mucins, focal adhesion pathway, ECM receptor interaction pathway

## Abstract

Endotoxins are toxic substances that widely exist in the environment and can enter the intestine with food and other substances. Intestinal epithelial cells are protected by a mucus layer that contains MUC2 as its main structural component. However, a detailed understanding of the mechanisms involved in the function of the mucus barrier in endotoxin penetration is lacking. Here, we established the most suitable proportion of Caco-2/HT-29 co-culture cells as a powerful tool to evaluate the intestinal mucus layer. Our findings significantly advance current knowledge as focal adhesion and ECM-receptor interaction were identified as the two most significantly implicated pathways in MUC2 small interfering RNA (siRNA)-transfected Caco-2/HT-29 co-culture cells after 24 h of LPS stimulation. When the mucus layer was not intact, LPS was found to damage the tight junctions of Caco-2/HT29 co-cultured cells. Furthermore, LPS was demonstrated to inhibit the integrin-mediated focal adhesion structure and damage the matrix network structure of the extracellular and actin microfilament skeletons. Ultimately, LPS inhibited the interactive communication between the extracellular matrix and the cytoskeleton for 24 h in the siMUC2 group compared with the LPS(+) and LPS(-) groups. Overall, we recognized the potential of MUC2 as a tool for barrier function in several intestinal bacterial diseases.

## Introduction

Bacterial endotoxins are toxic substances found on the cell walls of gram-negative bacteria. Lipopolysaccharide (LPS) is the main toxic substance of endotoxins, which are released by the death and rupture of gram-negative bacteria (1, 2). As endotoxins are more stable, they are widely distributed in a variety of environments. The human gastrointestinal tract has a large and complex array of commensal and harmful gram-negative bacteria that cannot damage the intestinal lumen when the intestinal mucosal barrier is intact (3, 4). However, when the intestinal mucosal immune barrier is damaged, many endotoxins translocate to the blood, causing endotoxemia. Ensuring the integrity of the intestinal mucosal barrier is thus key to preventing endotoxin translocation. The intestinal mucus layer shields host epithelial cells of the gastrointestinal tract from both normal microbiota and enteric pathogens (5–7). The main component of the intestinal mucus layer is MUC2 (mucin-2), which is produced by goblet cells and forms a highly organized glycoprotein network (8). The density of the mucus layer rapidly expands and attaches to the epithelial layer. Owing to the mucus layer, lumen bacteria, which are isolated from epithelial cells, cannot reach the surface of epithelial cells (9–11). Previous studies revealed that endotoxins do not damage intestinal epithelial cells in the presence of the mucus layer (12, 13). We hypothesized that the lack of the mucus layer is caused by the translocation of endotoxins to intestinal epithelial cells. However, the structures and functions of epithelial cells that are first damaged by endotoxins in the absence of the mucus layer are unclear. Here, a model of Caco-2 and HT-29 co-culture cells was established on 2D Transwell inserts to mechanistically investigate the endotoxin on the intestinal mucus layer based on intestinal barrier function. Further, the proliferation, structure, function, and mechanism of Caco-2/HT-29 cell co-culture after LPS treatment was evaluated using siRNA transfection analysis, RNA-seq, qPCR, ELISA, and immunofluorescence analysis. The differences in resource utilization reflect the regulatory mechanism of the endotoxin effect on the intestinal mucous barrier, providing insights into intestinal mucosal immune barrier function.

## Material and Methods

### Cell Culture and Cell Co-Culture

Caco-2 (BNCC Bio-350769, China) and HT-29 (BNCC Bio-350769, China) cells were seeded at density of of 1×10^5^ cells/cm^2^. Cells were grown in tissue culture flasks at 37°C, 5% CO_2_, and 90% relative humidity. All cell lines were cultured in DMEM-H medium (containing glutamine and sodium pyruvate) supplemented with 10% fetal bovine serum and 1% penicillin-streptomycin. Caco-2 and HT-29 cells were counted using a blood cell counter, mixed evenly in different proportions (Caco-2:HT-29 = 3:1 and 9:1), and seeded into the apical chambers of 24-well Transwell inserts (Corning, USA) at a final density of 1×10^5^ cells/cm^2^ in each insert. Cells were cultured in the same atmosphere described above, and allowed to grow for 15 days. The medium (500 μL in the upper compartment and 1500 μL in the lower compartment) was refreshed every other day. All samples were tested in six times repeat in this study (14, 15).

### Cell Viability Assay

The Cell Counting Kit-8 was used to quantify cell viability according to the manufacturer’s instructions. Briefly, 1 mL of a 3:1 (Caco-2: HT-29) cell suspension was seeded into 24-well plates at a density of 1×10^5^ cells/cm^2^ for 7 days. LPS was dissolved in DMEM-H and prepared in solutions of different concentrations (0, 100, 200, 400, and 800 µg/mL). LPS medium with different concentrations was added to a 24-well plate with 1 mL added to each well. Each dose group was assigned 24 wells. Six parallel wells for each dose group were exposed to LPS for 12 h, 24 h, 36 h, and 48 h. Blank wells were also established (no cells, only medium). The 24-well plates were removed at specific time points, the original culture medium. Thereafter, 100 µL of diluted CCK-8 reagent was added. After 1 h of incubation, the OD value of each well at a wavelength of 450 nm was measured using an automatic microplate reader (BioTek, USA); the measurement was repeated three times to calculate cell viability. Cell viability (%) =(OD value of experimental group OD value of blank group)/(OD value of control group OD value of blank group)×100.

### Alcian Blue/Periodic Acid-Schiff Stain

Acidic mucin and mucopolysaccharide produced by HT-29 cells in the control and LPS groups (400 µg/mL at 12 h, 24 h, 36 h, 48 h) were determined by Alcian blue and periodic acid-Schiff (PAS) staining. For Alcian staining, the cells were fixed with 4% paraformaldehyde for 30 min, soaked in Alcian acidification solution for 3 min, stained with Alcian staining solution for 30 min, and washed with running water. For PAS staining, the cells were fixed with PAS fixative (75% ethanol solution) for 10 min, oxidized in 1.0% periodic acid solution for 5 min in the dark, and then stained with Schiff’s reagent for 1 h at 37°C. After the addition of Schiff’s reagent, the samples were washed three times with sulfite solution for 1 min each. Nuclei were then stained with hematoxylin for 1 min, and the cells were imaged using an inverted microscope (Nikon, Japan).

### Immunofluorescence Analysis

The intercellular junctions of the LPS(-) and LPS(+) groups (400 µg/mL at 12 h, 24 h, 36 h, 48 h) were observed by immunofluorescence. The cells were seeded on glass coverslips, fixed for 30 min at 4°C with 4% paraformaldehyde, washed with PBS (three washes of 5 min each), permeabilized with 0.1% Triton-X-100 for 30 min at room temperature (RT), and rinsed with PBS at RT. To evaluate intercellular adhesion, cells were pre-incubated for 1 h with normal goat serum (5%, diluted in PBS) at RT to saturate non-specific binding sites. Incubation with the primary antibody, occludin (4°C overnight), and the secondary antibody, goat anti-rabbit IgG 488 (4 h at RT) was subsequently performed. Nuclei were stained with 10 g/mL DAPI for 5 min at RT. For each antibody, a technical negative control was used by replacing the primary antibody with PBS.

### ELISA

A total of 50 μL of the diluted standard was added to the standard wells and 40 μL of the diluent was added to each of the sample wells. Thereafter, 10 μL of the sample was mixed *via* gentle shaking, and 50 μL of the biotin antigen working solution was added. The plate membrane was covered, sealed, and then incubated for 1 h in a 37°C incubator. The sealing membrane was carefully removed, and the liquid was discarded. After the plate membrane was spin dried, each well was filled with washing solution, which was allowed to stand for 30 s. The solution was then discarded, and the process was repeated 5 times. Thereafter, the membrane was pat dried. A total of 50 μL of chromogen reagent A was added to the membrane, followed by 50 μL of chromogenic reagent B. After gentle mixing, the color was allowed to develop at 37°C for 15 min in the dark. Fifty μL of stop solution was then added to each well to stop the reaction, and the absorbance of each well was measured sequentially with a microplate reader at a wavelength of 450 nm.

### Small Interfering RNA Transfection

To prepare DNA-Hieff TransTM Liposome Nucleic Acid Transfection Reagent Complex, serum-free DMEM-H medium was mixed with MUC2 siRNA or negative control siRNA to a final concentration of 50 nM. Serum-free DMEM-H medium was also mixed with liposomal transfection reagent (liposomal transfection reagent: siRNA =3:1) and incubated for 3 min at RT. The diluted DNA and liposomal transfection reagent were gently mixed and incubated at RT for 20 min to form the DNA-liposome complex.

For cell transfection, 3:1 Caco-2 and HT-29 cells were seeded in 6-well plates at a seeding density of 1×10^5^ cells/cm^2^ before transfection. Thereafter, the co-cultured cells were transfected at a density of 90%-95% using antibiotic-free media plates. The DNA-Hieff Trans™ complex was added to each well of the cell culture plate, followed by incomplete DMEM-H medium to a total volume of 2 mL. The culture plate was then gently shaken and mixed. After 6 h of culture at 37°C in a 5% CO_2_ incubator, the growth medium was replaced. Further, after 48 h of culture, RNA was extracted, and the expression of the MUC2 gene in the transfection group and the LPS(-) group was detected by fluorescence quantitative PCR. FAM-negative control siRNA cells were transfected for 48 h and the transfection efficiency was evaluated under a fluorescence microscope.For challenged cells after transfection (LPS(+)+siRNA group), the transfection was performed for 48h according to the above steps. After 48h, the culture medium was discarded, washed with PBS twice, and 1.5mL medium containing 400 µg/mL LPS was added to continue culture for 24h in the incubator. For challenged cells after transfection (LPS(+)+siRNA group), the transfection was performed for 48h according to the above steps. After 48h, the culture medium was discarded, washed with PBS twice, and 1.5mL medium containing 400 µg/mL LPS was added to continue culture for 24h in the incubator.

MUC2 siRNA primer sequence F: 5`-GGAACAUGCAGAAGAUCAATT-3`

R: 5`-UUGAUCUUCUGCAUGUUCCTT-3`

FAM negative control sequence F: 5`-UUCUCCGAACGUGUCACGUTT-3`

R: 5`-ACGUGACACGUUCGGAGAATT-3`

### RNA-seq

The following three groups were established: LPS(-), LPS(+) (LPS 400 µg/mL, 24 h), and LPS(+)+siMUC2 (exposed to 400 µg/mL LPS for 24 h after transfection for 48 h). Total RNA was extracted from the three groups of cell samples, and the concentration, purity, and integrity of RNA were determined. mRNA was isolated from the total RNA, and double-stranded cDNA was synthesized. The adapter products were ligated, purified, and fragmented, and the final library was obtained using the Illumina TruSeq RNA Sample Prep Kit. High-throughput sequencing was then performed using the Illumina HiSeq 2500 system. The raw data were compared with the human genome, and differentially expressed genes were selected according to the criteria of |log2FC| ≥ 1 and p-value <0.05.

Occludin (*OCLN*), Claudin (*CLDN1*), *JAMA*, Desmosome, E-cadherin, and Zona occludens (*ZO1*) were selected as genes of tight-junction components. In addition, *FN1*, *ITGAV*, *ITGB3*, *COL6A2*, *HSPG2*, *LAMC1*, *LAMA5*, *LAMB2*, *CD44, DAG1*, and *SRC* genes in the ECM receptor interaction pathway, and *ITGB4*, *RhoA*, *ROCK1*, *ROCK2*, *ACTB-G1*, *ARHGAP5*, *ITGA2*, and *AGRN* in the focal adhesion pathway were selected. GAPDH was used as a housekeeping control.

### Real-Time PCR

RNA extraction and RT-PCR were performed according to previously published studies. Total RNA was extracted from the samples according to the instructions of the RNA extraction kit. Thereafter, the concentration, purity, and integrity of RNA were determined. The 5 All-In-one RT MasterMix kit (including additional gDNA removal steps) was used to reverse transcribe the RNA into cDNA according to the manufacturer’s instructions. Diluted cDNA was used as template DNA to assess gene expression levels. According to the human gene sequence, primers were designed using NCBI. The synthesized primers were then used for PCR amplification of the sample target fragment. RT-PCR was performed with an UltraSYBR Mixture on a fluorescence quantitative gene amplifier with three replicates per sample. GAPDH was used as a housekeeping gene to standardize the expression levels of the target genes. The relative expression levels of the target genes were calculated using the 2^-ΔΔCT^ method. All genes mRNA expression of LPS(-) group are “1” by analyse in the test.

**Table:** 

Gene	Primer name	Sequence	Product size(bp)
*MUC2*	F	ACCCGCACTATGTCACCTTC	151
	R	GGACAGGACACCTTGTCGTT	
*MUC5AC*	F	ACGGGAAGCAATACACGG	281
	R	GGTCTGGGCGATGATGAA	
*ALPi*	F	CCTGGTTGGGAAATAAGCACTC	136
	R	TTCAGAGGGAGGTCAGAAACAC	
*FN1*	F	GAGAATAAGCTGTACCATCGCAA	200
	R	CGACCACATAGGAAGTCCCAG	
*ITGAV*	F	GGCTGCATATTTCGGATTTTCTG	183
	R	CCATTCAGCTTTGTCGTCTGG	
*ITGB3*	F	AGTAACCTGCGGATTGGCTTC	164
	R	GTCACCTGGTCAGTTAGCGT	
*COL6A2*	F	AGCCTACGGAGAGTGCTACA	173
	R	GTCCTGGGAATCCAATGGGG	
*LAMC1*	F	CTGCAAAGAAGGGACGGGAT	139
	R	ATGGTCTGGTTGATGGCAGG	
*LAMA5*	F	GGGGTGTCTGTATCGACTGC	204
	R	ACCGCTCCCCAGAGAAGTT	
*LAMB2*	F	GGAACGCTCAGCAGACTTTG	131
	R	AGCGGGACTCACAGACTACAT	
*CD44*	F	CTGCCGCTTTGCAGGTGTA	109
	R	CATTGTGGGCAAGGTGCTATT	
*ARGN*	F	GACTTCAACGGCTTCTCCCA	134
	R	TTCTGCCCGTTGTAGAGCAG	
*HSPG2*	F	CCAAATGCGCTGGACACATTC	206
	R	CGGACACCTCTCGGAACTCT	
*DAG1*	F	TCAAGGCCAAGTTTGTGGGT	139
	R	GAGCCCCGGGTGATATTCTG	
*ITGA2*	F	GGGAATCAGTATTACACAACGGG	112
	R	CCACAACATCTATGAGGGAAGGG	
*ITGB4*	F	TGTCCATCCCCATCATCCCT	106
	R	CCCGATGGAGAGCGTAGAAC	
*SRC*	F	TGGCAAGATCACCAGACGG	100
	R	GGCACCTTTCGTGGTCTCAC	
*ARHGAP5*	F	ACCGAAGGACTCTACCGTGT	211
	R	CCGGGATTTTTGCTGCTTCC	
*RhoA*	F	GAGCCGGTGAAACCTGAAGA	146
	R	TTCCCACGTCTAGCTTGCAG	
*ROCK1*	F	CCAATTGTGATGCCTGTGCC	281
	R	AGAAAGCGTTCGAGGGGAAG	
*ROCK2*	F	AGTTGGTTCGTCACAAGGCA	207
	R	CTCCACCAGGCATGTACTCC	
*ACTB-P1*	F	CATGTACGTTGCTATCCAGGC	250
	R	CTCCTTAATGTCACGCACGAT	
TPJ1	F	ACCAGTAAGTCGTCCTGATCC	128
	R	TCGGCCAAATCTTCTCACTCC	
CLDN1	F	GAGGTGCCCTACTTTGCTGT	102
	R	ACACGTAGTCTTTCCCGCTG	
F11R	F	CCAAGGAGACACCACCAGAC	184
	R	GAGCTTGACCTTGACCTCCC	
DSC3	F	GGAGGGCAGGAAACCATTGA	94
	R	TGCAGGAGTCCAGGGTATGA	
OCLD	F	AGCAGCGGTGGTAACTTTGA	113
	R	CCGCCAGTTGTGTAGTCTGT	
*GAPDH*	F	CATCAAGAAGGTGGTGAAGCAG	120
	R	GCGTCAAAGGTGGAGGAGTG	

### Statistical Analysis

Statistical analyses were performed using GraphPad Prism 7, and the data are expressed as mean ± SD. Statistical analyses were performed using the Statistical Package for Social Sciences (SPSS, USA) software, and a two-pair test followed by Student’s t-test was performed to determine statistical significance between means. * Statistical difference between co-culture Caco2/HT-29 cells in the LPS(+) and LPS(+)+siMUC2 groups for the same factors (*P* < 0.05).

## Results

### Determination of the Optimal Mucus Layer of the Caco-2/HT-29 Co-Culture Model in Transwell

**Figure 1:**
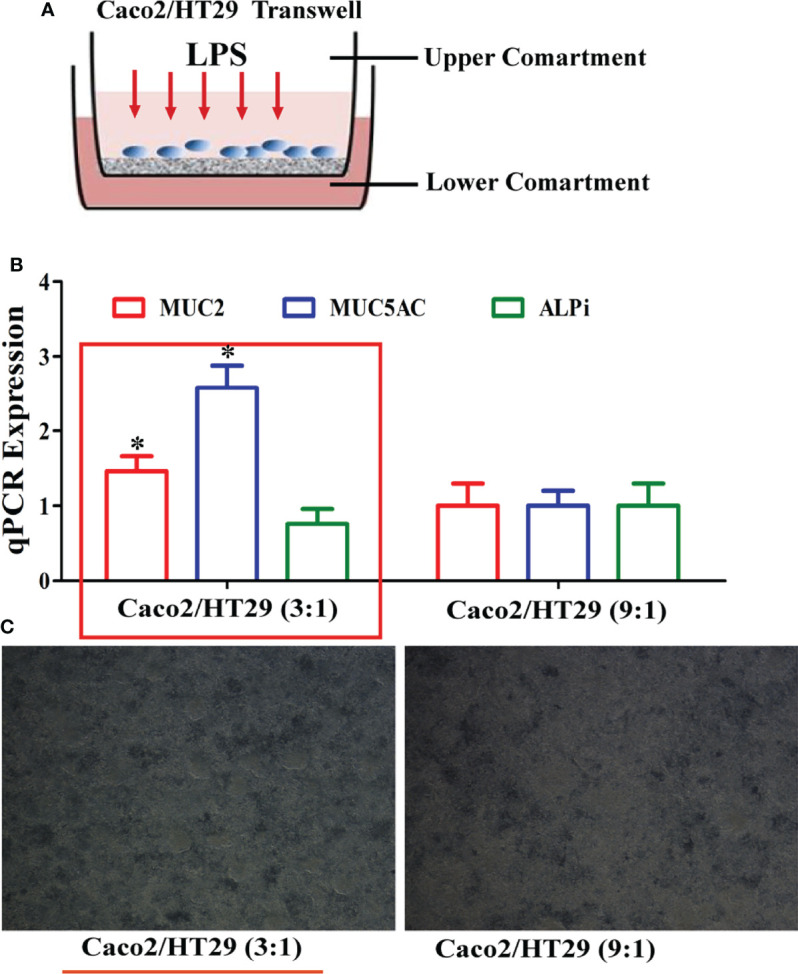
Establishment of the Caco2/HT-29 cell co-culture model. **(A)**, Transwell of the Caco-2/HT-29 co-culture cell model; **(B)**, MUC2, MUC5AC, and ALPi mRNA expression of the Caco-2/HT-29 co-culture cell at two cell seeding ratios (N=6); **(C)**, Image of the Caco-2/HT-29 co-culture cell at two cell seeding ratios (×200).* Statistical difference between co-culture Caco2/HT-29 cells (3:1) compare to co-culture Caco2/HT-29 cells (9:1) for the same factor (*P*<0.05).

HT-29 cells are often used as a goblet cell model *in vitro*, and can produce mucin secretions, forming an extracellular mucus layer. Herein, the combination of Caco-2 and HT-29 co-culture cells in the transwell compartment was selected to ensure that the established intestinal epithelial model had a mucus layer; this was determined by measuring epithelial monolayer integrity and mucus production (**Figure 1A**). In other studies, Caco-2:HT-29 ratios of 9:1 and 3:1 showed similar results; the TEER values also reached 400 Ω×cm^2^ at the end of the 15-day differentiation process (14). Therefore, at the end of 15 days of differentiation, the optimal Caco-2: HT-29 ratios were 3:1 and 9:1, respectively. ALPi is a marker of Caco-2 cell differentiation, MUC5AC is a marker of HT-29 cell differentiation, and MUC2 is the main component that forms the mucus layer skeleton. Here, MUC2 and MUC5AC mRNA expression in Caco-2/HT-29 (3:1) co-culture markedly increased relative to that in the Caco-2/HT-29 (9:1) co-culture at the end of the 15-day differentiation process (**Figures 1B, C**). These combined data indicate that the Caco-2:HT-29 ratio of 3:1 led to the best mucus barrier at the end of the 15-day differentiation in the Transwell compartment.

### PAS/Alcian Blue Staining and Immunofluorescence Analysis of Mucus Secretion and Occludin Expression in Caco-2/HT-29 Co-Culture Cell

**Figure 2:**
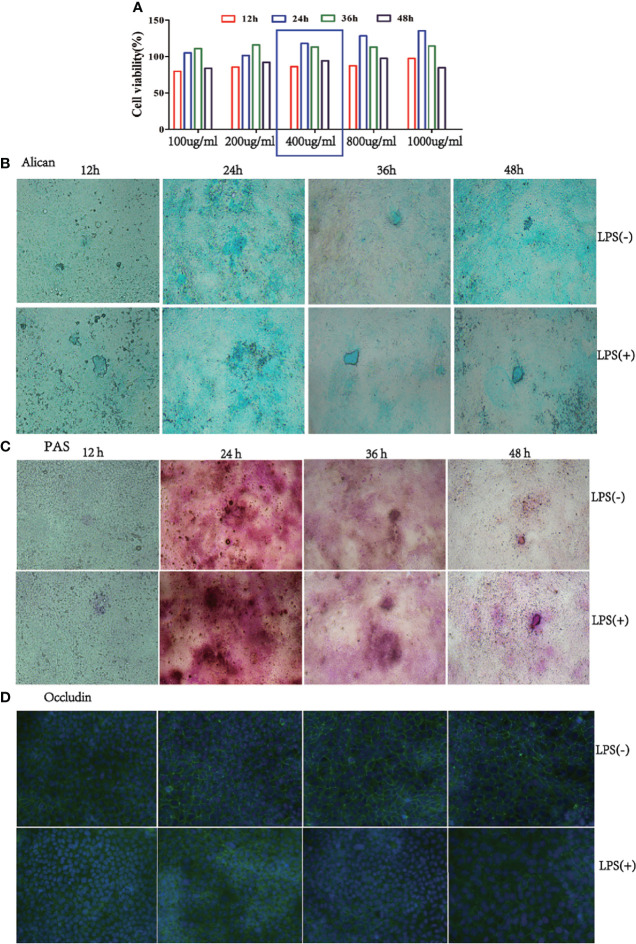
Mucus expression in the co-culture model at a 3:1 ratio of Caco-2/HT-29 cells. **(A)**, Viability of the Caco-2/HT-29 co-culture cell (3:1) was determined using CCK-8 after stimulation with 100, 200, 400, 800, and 1000 µg/mL LPS for 12 h, 24 h, 36 h, and 48 h (N=6); **(B)**, Alcian Blue staining of Caco-2 and HT-29 co-culture cell stimulated with LPS for 12 h, 24 h, 36 h, and 48 h. Blue areas indicate mucus deposition (Nikon Plan 10× objective lens); **(C)**, Periodic Acid-Schiff staining (PAS) on Caco-2/HT-29 co-culture cell stimulated with LPS for 12 h, 24 h, 36 h, and 48 h. Fuchsia areas indicate mucus deposition (Nikon Plan 10× objective lens); **(D)**, Occludin expression of Caco-2/HT-29 co-culture cell was analyzed by immunofluorescence. The green color represents the occludin expression of Caco-2 and HT-29 co-culture cells; blue color indicates the nucleus of Caco-2/HT-29 co-culture cell (×100).

To confirm the effect of the best time and dose of LPS on the mucus barrier function, we used 100, 200, 400, 800, and 1000 µg/mL LPS to stimulate Caco-2/HT-29 (3:1) co-culture cells at 12, 24, 36, and 48 h, respectively, after 15 days of Caco-2/HT-29 (3:1) co-culture differentiation. Cell viability was found to be highest after 24 h of LPS stimulation; thereafter, cell viability began to decline. Stimulation of Caco-2/HT29 (3:1) co-culture cells with 800 and 1000 µg/mL LPS caused cell death at 48 h. Therefore, 400 µg/mL LPS was selected to stimulatCaco-2/HT-29 (3:1) co-culture cells in the next test (**Figure 2A**). Based on PAS and Alcian Blue staining, mucin secretion by Caco-2/HT-29 cell was significantly increased at 24 h compared with 36 h and 48 h in the LPS(-) group. Further, the LPS(+) group displayed remarkable increases in mucin secretion compared with LPS(-) group after LPS stimulation for 12, 24, 36, and 48 h (**Figures 2B, C**). Using immunofluorescence, we evaluated the expression of occludin transmembrane proteins in Caco-2/HT-29 (3:1) co-cultured cells at different time points to assess the presence of tight junctions (**Figure 2D**). Caco-2/HT-29 cells in the LPS(-) group had good cell membrane connectivity at 24, 36, and 48 h. In contrast, the connection of the cell membrane for LPS stimulation was damaged in the LPS(+) group at 24, 36, and 48 h. Taken together, these results indicate that mucin secretion and occludin expression in Caco-2/HT29 (3:1) co-culture cells markedly increased in the LPS(-) and LPS(+) groups after 24 h of LPS stimulation.

### RNA Interference for MUC2 Gene Silencing in Caco-2/HT-29 (3:1) Co-Culture Cell

**Figure 3:**
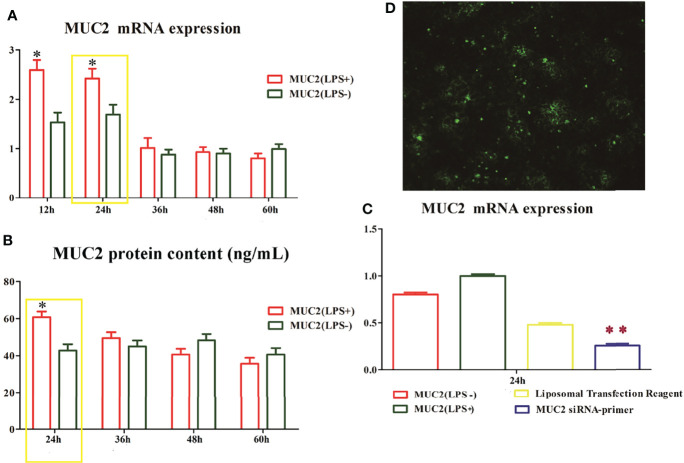
The time and dose screen of small RNA interference. **(A)**, MUC2 mRNA expression in Caco-2/HT-29 co-culture cells after LPS stimulation for 12 h, 24 h, 36 h, 48 h, and 60 h; The highest level of MUC2 mRNA expression in Caco-2/HT-29 co-culture cells is observed at 24 h after LPS stimulation (N=6); **(B)**, MUC2 protein content in the supernatant of Caco-2/HT-29 co-culture cells after LPS stimulation for 24 h, 36 h, 48 h, and 60 h (N=6); the highest level of MUC2 protein content in Caco-2/HT-29 co-culture cells is observed at 24 h after LPS stimulation; * Statistical difference between co-culture Caco2/HT-29 cells in the LPS(+) compare to LPS(-) groups for the same time (*P*<0.05); **(C)**, After LPS stimulation for 24 h, the Caco-2/HT-29 co-culture cells were transfected with 50 nM MUC2 siRNA or 50 nM negative control siRNA (siNC) using Liposomal Transfection Reagent. The MUC2 mRNA expression of MUC2 siRNA group was significantly decreased compare with that of the MUC2(LPS+) and MUC2(LPS-) groups (N=6); ** Statistical difference between co-culture Caco2/HT-29 cells in the MUC2 siRNA-primer compare to LPS(+) and LPS(-) groups (*P <*0.01); **(D),** Small interfering RNA transfection rate, the green point indicates positive signal of MUC2 small interfering RNA transfection.

To understand the function of the mucus layer, we used RNA interference (RNAi) to silence the *MUC2* gene (the main component of the mucus layer) in Caco-2/HT-29 (3:1) co-cultured cells. First, we evaluated MUC2 mRNA and protein expression using qPCR and ELISA, respectively. After 15 days of Caco-2/HT-29 (3:1) co-culture differentiation, cells were stimulated with LPS(LPS+) or 0.0 1M PBS (LPS-) at 24, 36, 48, and 60 h. The expression levels of MUC2 mRNA and protein in the LPS(+) and LPS(-) groups were higher at 24 h than at other time points. Therefore, Caco-2/HT-29 (3:1) co-culture cells were transfected with siRNA MUC2 constructs using Lipofectamine ™ 2000 **before** stimulation with LPS for 24 h (**Figures 3A, B**). MUC2 mRNA expression was significantly decreased after transfection with MUC2 siRNA. A green fluorescent negative control was used to determine the transfection efficiency (**Figures 3C, D**). These combined results indicate the significant increase in MUC2 mRNA expression after transfection with siRNA and LPS stimulation for 24 h. All co-cultured cells were divided into three groups, namely LPS (-), LPS (+), and siMUC2 +LPS(+) in the following test.

### Analysis of the Different Genes and KEGG Enrichment Pathways for the LPS (-), LPS (+), and siMUC2 +LPS(+) Groups by RNA-seq

**Figure 4:**
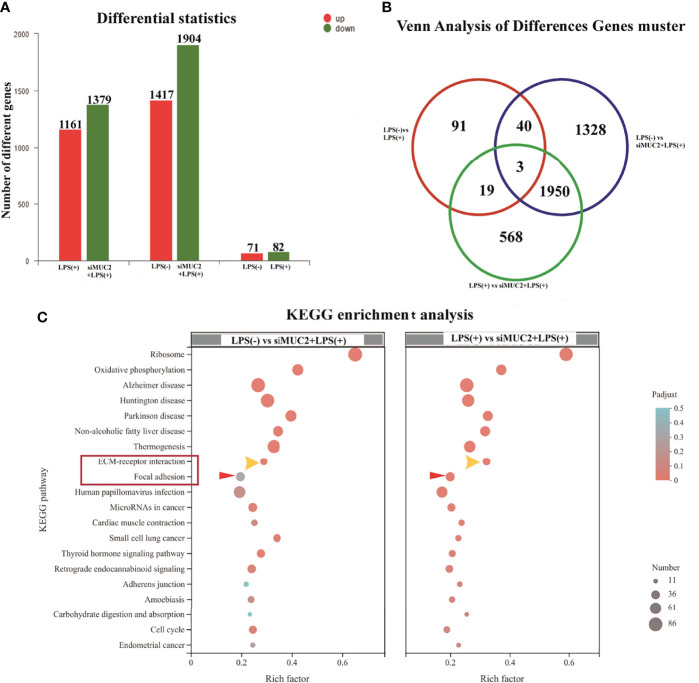
RNA-seq of the LPS(+) group, LPS(-) group, and siMUC2+LPS(+) group. **(A),** the number of different genes in the LPS(+) vs siMUC2+LPS(+) group, LPS(-) vs siMUC2+LPS(+) group, and LPS(-) vs LPS(+) group; red color indicated upregulated gene and blue color indicates downregulated gene (N=6); **(B)**, Venn analysis of different gene clusters in the LPS(+) vs siMUC2+LPS(+) group, LPS(-) vs siMUC2+LPS(+) group, and LPS(-) vs LPS(+) group; Three genes were found between the LPS(+) vs siMUC2+LPS(+) group, LPS(-) vs siMUC2+LPS(+) group, and LPS(-) vs LPS(+) group; 43 genes were found between the LPS(-) vs siMUC2+LPS(+) group and LPS(-) vs LPS(+) group; 22 genes were found between the LPS(-) vs LPS(+) group and LPS(+) vs siMUC2+LPS(+) group; and 1,953 genes were found between the LPS(+) vs siMUC2+LPS(+) group and LPS(-) vs siMUC2+LPS(+) group (N=6); **(C),** KEGG enrichment analysis of the LPS(+) vs siMUC2+LPS(+) group (right column) and LPS(-) vs siMUC2+LPS(+) group (left column); the first 20 signaling pathways with differentially expressed genes are listed in the figure.

To provide insights into how the regulatory mechanism of LPS affects the mucus layer, the different genes and KEGG enrichment pathways in the LPS (-), LPS (+), and siMUC2 +LPS(+) groups were analyzed using RNA-seq techniques. A total of 1,161 upregulated genes and 1,379 differentially expressed genes were found in the LPS (+) and siMUC2 +LPS(+) groups. Further, 1,417 and 1,904 genes were upregulated and downregulated, respectively, in the LPS (-) vs. siMUC2 +LPS(+) group, and 71 different genes were upregulated and 82 genes were downregulated in the LPS (-) vs. LPS(+) group (**Figure 4A**). A total of 1,953 differentially expressed genes were found between the LPS (-) vs siMUC2+LPS group and LPS (+) vs siMUC2 +LPS group (**Figure 4B**). The first 20 signaling pathways were measured by KEGG enrichment analysis between the LPS (-) vs. siMUC2+LPS(+) group and LPS (+) vs. siMUC2+LPS group (**Figure 4C**). The cell extracellular matrix (ECM) receptor interaction and focal adhesion signaling pathways were used to further study the mechanism of mucus layer function.

### Expression of Different Genes in ECM-Receptor Interaction and the Focal Adhesion Signal Pathway and Intercellular Linker

**Figure 5:**
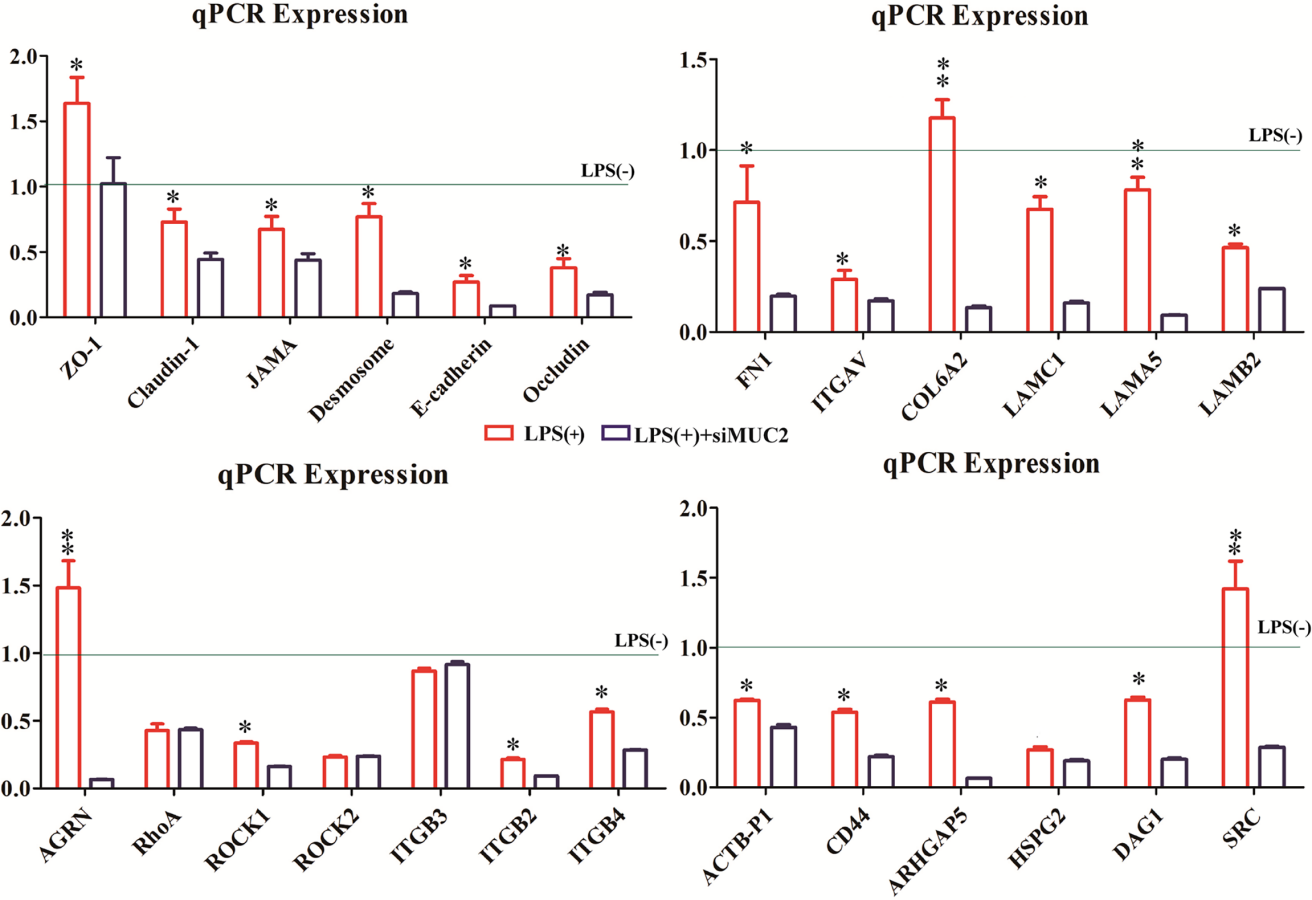
The expression of different genes in the LPS(+), LPS(+)+siMUC2, and LPS(-) groups.Statistical analyses were performed using the Statistical Package for Social Sciences (SPSS, USA) software, and a two-pair test followed by Student’s t-test was performed to determine statistical significance between means (N=6). The relative expression levels of the target genes were calculated using the 2^-ΔΔCT^ method. The factors mRNA expression of LPS(-) group are “1” (gray line).* Statistical difference between co-culture Caco2/HT-29 cells in the LPS(+) and LPS(+)+siMUC2 groups for the same factors (*P*<0.05). ** Statistical difference between co-culture Caco2/HT-29 cells in the LPS(+) and LPS(+)+siMUC2 groups for the same factors (*P <*0.01).

To understand the different genes involved in ECM-receptor interactions and the focal adhesion pathway, we measured the mRNA expression of these factors in the LPS (+) and siMUC2+LPS(+) groups using qPCR. After 15 days of Caco-2/HT-29 (3:1) co-culture differentiation, the cells were stimulated with LPS for 24 h, and total RNA was extracted from cells in the LPS (+) and siMUC2+LPS(+) groups. The mRNA expression levels of Claudin-1, ZO-1, JAMA, Desmosome, Occludin, and E-cadherin were significantly increased in the LPS (+) group compared with the siMUC2 +LPS group (*P*<0.05). Further, the mRNA expression levels of FN1, ITGAV, COL6A2, LAMC1, LAMA5, LAMB2, AGRN, ROCK1, ITGB2, ITGB4, ACTB-P1, CD44, ARHGAP5, HSPG2, DAG1, and SRC were significantly increased in the LPS (+) group, compared with siMUC2 +LPS(+) group (*P*<0.05). There was no difference in the mRNA expression of ITGB3, ROCK2, and RhoA between the LPS (+) group and siMUC2 +LPS(+) group (*P*>0.05) (**Figure 5**).

## Discussion

The combination of Caco2/HT-29 cells is an already-described *in vitro* epithelial barrier model in the real environment of the human intestine, and has been proposed as a predictive tool to evaluate the permeability of endotoxins (16–18). In this study, we employed the co-culture model and identified that 3:1 (Caco-2:HT-29) was the optimal ratio for cell seeding compared to 9:1 (Caco-2:HT-29) after 15 days of Caco-2/HT-29 co-culture. The use of a wide set of gene markers (MUC5AC and ALPi) was also found to be appropriate for evaluating the integrity of the co-culture cell model. The mRNA expression of MUC2 corresponded to the *in vivo* human colonic epithelium after 15 days of Caco-2/HT-29 co-culture. Herein, 400 µg/mL LPS was selected to stimulate co-cultured cells for 12 h, 24 h, 36 h, and 48 h.

To choose a time that will ensure that Caco-2 and HT-29 cells have a well-established mucus shed, Alcian Blue and PAS staining was performed on the co-cultured cells. Mucin secretion was identified to be the highest in the LPS(+) and LPS(-) group after 24 h. Further, confocal microscopy was verified as a powerful tool for visualizing the tight junctions of cells in these barriers. After 15 days of Caco-2/HT-29 co-culture, the tight junction function of co-culture cells in the LPS(-) group was found to be better after 24 h of PBS stimulation; however, the tight junction function of co-culture cells in the LPS(+) group was damaged by LPS stimulation for 24 h. Additionally, the mRNA and protein expression levels of MUC2 were the highest following LPS stimulation for 24 h relative to the other times investigated. Taken together, after 15 days of Caco-2/HT-29 co-culture, the highest value of MUC2 secretion was obtained 24 h after LPS stimulation.

Based on the above results, we opted to silence the MUC2 gene in the co-culture cells *via* small interfering RNA transfection for LPS stimulation at 24 h after 15 days of Caco-2/HT-29 co-culture. MUC2 mRNA expression was significantly inhibited. Therefore, the co-cultured cells were divided into three groups (LPS(+), LPS(-), and LPS(+)+siMUC2 groups) for the subsequent experiment. The usefulness of RNA-seq to demonstrate the effect of LPS on the mucus layer in an *in vitro* intestinal model was evaluated. A total of 1,161 upregulated genes and 1,379 downregulated genes were found in the LPS(+) vs. LPS(+)+siMUC2 group; 1,417 upregulated genes and 1,904 downregulated genes were found in the LPS(-) vs. LPS(+)+siMUC2 group; and 71 upregulated genes and 82 downregulated genes were found in the LPS(+) vs. LPS(-) group. LPS stimulation for 24 h led to very few differential genes that had an impact on the integrity of the mucus barrier of Caco-2/HT-29 co-culture cells. Further, LPS did not disrupt the mucus barrier to penetrate the epithelial cells. Nonetheless, significant changes in a large number of genes were found in the LPS(-) and LPS(+) groups when the MUC2 gene was silenced by LPS stimulation for 24 h. The intestinal mucus layer may primarily act as a barrier that protects epithelial cells from LPS stimulation (19–22).

**Figure 6:**
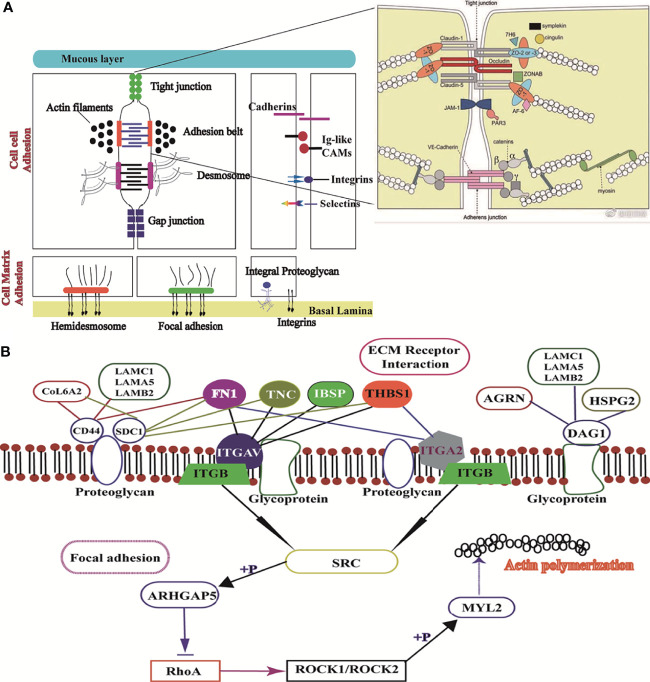
Image of ECM receptor interaction and the focal adhesion signaling pathway. **(A),** Structure of the mucus layer, cell-cell adhesion, and cell-matrix adhesion of intestinal epithelial cells; **(B)**, Interaction mechanism of the difference factor for ECM receptor interaction and the focal adhesion signaling pathway.

From top to bottom, the complete intestinal mucosal barrier is composed of the mucus layer, epithelial cell layer, and muscle layer (23–25). Further, the mucins of the mucus layer are fused with water and digestive juice in the intestinal tract, which together form the first barrier of intestinal mucosa against pathogenic microbial and toxic substances (26). Both the junctional network and the mucous layer protect the integrity of the intestinal epithelium (27). The intestinal mucus layer is a mechanical barrier comprising intestinal epithelial cells and various intercellular connections (28). Intestinal epithelial integrity is dependent on the organization of cell-cell adhesion and cell matrix adhesion complexes, including occluding junctions, anchoring junctions, and communication junctions (29). The most important way of occluding junctions is tight junctions (TJs), which include claudins and occludins, which interact with each other on their extracellular sides to promote junction assembly (30). In this study, the mRNA expression levels of claudins, JAMA, E-cadherin, and occludin were significantly increased in the LPS(+) group compared to the LPS(+) +siMUC2 group. E-cadherin is the most essential cadherin present on the epithelial surface and is responsible for the formation of adhesion junctions. E-cadherin hinges on the neighboring cell through another E-cadherin. *via* Based on our results, LPS disrupts the tight junctions and penetration barriers in epithelial cells after MUC2 gene silencing in co-culture cells (**Figure 6A**).

LPS acts on the anchoring junction, which mainly connects with intermediate fibers such as desmosomes and hemidesmosomes, through tight junctions (31–33). Further, the anchoring junction connected with actin fibers mainly includes focal adhesion and adhesion belts.In this study, desmosome mRNA expression was significantly higher in the LPS(+) group than in the LPS(+) +siMUC2 group. Using the differential gene lists, focal adhesion and ECM-receptor interaction were identified as the two most significantly implicated pathways by KEGG analysis. Cell-ECM interactions are crucial for cell survival and normal cellular functions, such as cell adhesion, spreading, and migration, and regulate the establishment and maintenance of development and homeostasis (34). ECM is a complex network structure composed of cell synthesis and biomacromolecules on the cell surface or between cells. As the ECM-receptor interactions play a critical role in focal adhesion, genes related to ECM-mediated focal adhesion are of particular interest as potential transcriptomic markers of the intestinal mucus layer (35). In addition to controlling cell movement and migration, focal adhesions physically adhere to the external environment by attaching themselves to the ECM (36). In our study, gene expression related to ECM-mediated focal adhesion was mixed, and adhesion glycoproteins (THBS1), fibronectin (FN1), and other ECM molecules can directly and indirectly bind to cell surface receptors. The ECM, through cell surface receptors, transmits extracellular to intracellular signals, which together affect cell function. Integrins are the main receptors for ECM proteins. Integrins (ITGAV, ITGB3,4 and ITGA2) of cell surface receptors are used as “bridges” to connect ECM, and the intracellular cytoskeleton formed an organic body, mediating intracellular signal transduction (37). In our experiment, the expression levels of THBS, IBSP, and FN1 were downregulated in the LPS(+)+siMUC2 group compared to the LPS(+) group. they are high affinity ligand for ITGB4, ITGAV, ITGA2 of cell membrane, were found to be downregulated in the co-culture cell. Integrins can activate SRC kinase; however, SRC expression in the LPS(+)+siMUC2 group was downregulated compared with that in the LPS(+)group, and was inhibited downstream of the ECM-mediated focal adhesion pathway. SRC is specifically phosphorylated and inhibited by ARHGAP5 and RhoA expression (38). Coiled-coil-forming protein kinase (ROCK1,2) downstream of RhoA is well known for its inability to regulate the stability of filamentous actin (ACTB-G1) in the LPS(+) +siMUC2 group compared to the LPS(+) group (**Figure 6B**). Actin provides mechanical support to cells and a transport pathway through the cytoplasm to assist with the rapid assembly and disassembly of the signal transduction actin network, enabling cell migration. Cells connect the ECM network to the intracellular actin microfilament skeleton through integrin-mediated focal adhesion structures (39). The extracellular mechanical force activates SRC kinase through focal adhesions and promotes further maturation of the focal adhesion structure. Further, the intracellular mechanical force generated by actin contraction is transmitted to the focal adhesion through adaptor proteins. Actin contraction drives the movement of integrins along the microfilament cytoskeleton, from focal adhesions at the cell edge to fibrillar adhesions in the middle of the cell body (40). According to our results, when only the ECM integrin-mediated focal adhesion-action is connected, integrins play a role in signal transduction. When the connection between the adaptor protein and actin is disrupted, integrin slides on the surface of the cell membrane without signal transmission.

This study also sought to elucidate the usefulness of KEGG to demonstrate the impact of LAMB2, COL6A2, and FN1 downregulation on ECM binding to CD44 of proteoglycan receptor on the cell membrane. The downregulation of CD44 in the LPS(+)+siMUC2 group was found to inhibit the proteoglycan of the cell membrane. The ECM membrane receptor, CD44, can integrate ECM signals and regulate cell adhesion, migration, and proliferation (31). LAMB2 and AGRN bind to DAG1 and promote glycoprotein secretion by the cell membrane (40). However, when the MUC2 gene was silenced in the co-culture cells, the decrease in glycoprotein secretion of the ECM due to LAMB2, AGRN, and DAG1 mRNA expression was reduced (**Figure 6B**). Transmembrane-linked glycoproteins of mesenchymal cells, which are linked to attachment proteins in the intracellular portion, interact with transmembrane-linked glycoproteins of adjacent cells or the ECM in the extracellular portion (41). Cell surface glycoproteins are membrane surface glycoproteins that mediate adhesion between cells, and between cells and the ECM (39). These glycoproteins influence the spatial organization and function of the transmembrane receptors. A small amount of glycoproteins and proteoglycan inhibited the adhesion of integrins and altered the integrin state by applying tension to matrix-bound integrins, independent of actomyosin contractility (41).

## Conclusion

In summary, in addition to the data that enabled the establishment of the proposed Caco-2/HT-29 model, we defend its use as a powerful tool for evaluating the intestinal mucus barrier. LPS (400 µg/mL) was found to disrupt the regulatory mechanism of the ECM-mediated focal adhesion signaling pathway under MUC2 gene silencing in co-cultured cells after 24 h of LPS stimulation. When the mucus layer is not intact, LPS first damages the tight junctions of epithelial cells, regulates the integrin of cell surface receptors through the ECM transmitted to downstream signals, and inhibits the integrin-mediated focal adhesion structure, further damaging the ECM network structure and intracellular actin microfilament skeleton. Ultimately, LPS inhibits the interaction between the ECM and cytoskeleton. By combining these data, the protective function of the mucus barrier is expected to be well characterized in the future.

## Data Availability Statement

The original contributions presented in the study are included in the article/supplementary material. Further inquiries can be directed to the corresponding author.

## Author Contributions

CW and PF conceived and designed the experiments. WH, MZ, YL and ShuW performed the experiments. WH and TT analyzed the data and wrote the manuscript. All authors contributed to the article and approved the submitted version.

## Funding

This study was supported by the Shaanxi province Key research projects (2020NY-020) and the National Natural Science Foundation of Shaanxi (K4030220016).

## Conflict of Interest

The authors declare that the research was conducted in the absence of any commercial or financial relationships that could be construed as a potential conflict of interest.

## Publisher’s Note

All claims expressed in this article are solely those of the authors and do not necessarily represent those of their affiliated organizations, or those of the publisher, the editors and the reviewers. Any product that may be evaluated in this article, or claim that may be made by its manufacturer, is not guaranteed or endorsed by the publisher.

## Abbreviations

FN1, Fibronecin; ITGAV, integrin subunit alpha V; ITGB3, integrin beta 3; COL6A2, Collagen alpha-2(VI) chain VI; HSPG2, heparan sulfate proteoglycan 2; LAMC1, laminin subunit gamma 1; LAMA5, laminin subunit alpha 5; LAMB2, laminin subunit beta 2; CD44, CD44 molecule (Indian blood group); DAG1, dystroglycan 1; SRC, SRC proto-oncogene, non-receptor tyrosine kinase; ITGB4, integrin subunit beta 4; RhoA, ras homolog family member A; ROCK1, Rho associated coiled-coil containing protein kinase 1; ROCK2, Rho associated coiled-coil containing protein kinase 2; ACTB-G1, actin beta/gamma 1; ARHGAP5, Rho GTPase activating protein 5; ITGA2, integrin subunit alpha 2; AGRN, Agrin.
